# From Crisis to Victory: Rescuing Root Canal Treatment With Bioactive Material in a Nail-Biting Case of Furcal Perforation

**DOI:** 10.7759/cureus.60414

**Published:** 2024-05-16

**Authors:** Tejas Suryawanshi, Manoj Chandak, Aditya Patel, Anuja Ikhar, Paridhi Agrawal, Palak Hirani

**Affiliations:** 1 Conservative Dentistry and Endodontics, Sharad Pawar Dental College and Hospital, Datta Meghe Institute of Higher Education and Research, Wardha, IND; 2 Conservative Dentistry and Endodontics, Sharad Pawar Dental College, Datta Meghe Institute of Higher Education and Research, Wardha, IND

**Keywords:** treatment outcomes, furcal perforation repair, fiber-reinforced composites (frcs), mineral trioxide aggregate (mta), endodontic perforation

## Abstract

This case report illustrates the successful treatment of an iatrogenic furcal perforation using mineral trioxide aggregate (MTA) and its reinforcement with fiber-reinforced composites (FRCs). Endodontic perforations, particularly in the furcal area, present unique challenges that demand effective management strategies. MTA, known for its multifaceted properties including osteoinductive activity and sealing capabilities, has emerged as the gold standard material for perforation repair. This case report demonstrates the application of MTA in repairing the perforation site following thorough cleaning and shaping of the root canals. Furthermore, the use of FRCs, specifically glass fiber-reinforced composite (GFRC), is introduced to reinforce the repaired site, enhancing its mechanical properties and long-term stability. The discussion emphasizes the importance of selecting appropriate materials for endodontic perforation repair and highlights the advantages of FRCs in preventing structural failures. Future directions suggest further research to refine FRC formulations and standardize clinical protocols to maximize treatment outcomes. This case report contributes valuable insights to the advancement of endodontic therapy, showcasing the combined benefits of MTA and FRCs in achieving favorable treatment outcomes for iatrogenic furcal perforations.

## Introduction

Bioactive material has earned a reputation as the gold standard material for the repair of perforation in endodontic procedures over the years. Extensive research, including studies conducted by Thomson et al., has shed light on its multifaceted properties and applications in dental therapy [1]. Thomson et al. investigated mineral trioxide aggregate (MTA)'s role in enhancing osteocalcin synthesis and stimulating mineralized matrix production, indicating its potential as a cement conductor and osteoinductor material [1]. Their findings underscored the significant contribution of MTA in promoting tissue repair and regeneration, crucial aspects in the success of endodontic treatments.

Moreover, Moretton et al. delved into MTA implantation in osseous tissue and subcutaneous conjunctival tissue of rats, unveiling its remarkable osteoinductive activity [2]. This study further solidified MTA's position as a versatile material capable of eliciting favorable biological responses crucial for tissue healing. Beyond its osteoinductive properties, MTA's physical sealing capabilities have garnered considerable attention. With its insolubility in the presence of blood and the release of calcium ions contributing to its high pH, MTA acts as an effective barrier against bacterial invasion, safeguarding the integrity of dental structures and facilitating the repair process [2,3].

MTA has earned widespread recognition as an excellent material for various endodontic treatments, including repair of perforation, filling of retrograde, and vital pulp therapy. The main constituents of MTA include several mineral oxides, which dictate its chemical and physical properties. MTA is a hydrophilic mineral powder of various oxides. It sets in contact with water or moisture within three to four hours. Numerous studies have exhibited its outstanding ability to seal and biocompatibility. Through different leakage assessment methods such as fluid filtration, dye-leakage models, bacterial leakage models, and extraction of dye techniques, MTA has exhibited better-sealing properties in contrast to other materials like amalgam, zinc oxide-eugenol cement, and resin-modified glass ionomer cements (RMGICs).

Moreover, MTA possesses the desirable characteristics of being biocompatible, nonabsorbable, radiographically opaque, and bacteriostatic or bactericidal, making MTA an ideal material for treating radicular perforations. The reparative capacity of MTA can be attributed to its bacteriostatic and bacteriocidal properties and high pH, which promote cementum growth and bone formation.

Iatrogenic perforation is an undesirable event that can occur during endodontic procedures, such as access cavity preparation or exploration of canal orifices. Such incidents may result from excessive excavation of dentin while attempting to locate canals or due to the failure to achieve straight-line access. Perforations may occur laterally or through the chamber floor into the furcal area. After some accidental perforations, surgical management or sacrifice of tooth may be the only two treatment modalities.

Despite the prevalence of furcal perforations, there have been relatively few reports on their repair using MTA. However, several studies have documented successful clinical results with application of MTA in repairing furcal perforations. For instance, Arens and Torabinejad described two cases in which MTA effectively repaired furcation perforations [4]. Additionally, Pace et al. conducted long-term clinical and radiographic follow-ups, indicating successful sealing of perforations in the majority of cases [5]. Oliveira et al. reported successful repair of furcal perforations with MTA in primary molar teeth, with favorable clinical and radiographic outcomes [6]. Similarly, Silveira et al. documented successful cases of furcation perforation repair using MTA [7], but very limited literature is present on its reinforcement for function. The objective of this article is to present a clinically viable approach for treating large perforation using a bioactive material and its reinforcement.

## Case presentation

A 52-year-old female visited the Department of Endodontics, Sharad Pawar Dental College and Hospital, after a primary appointment one week prior at a private dental clinic at Hinganghat for Endodontic treatment of tooth number 26. She reported that during the endodontic procedure, the treating dental surgeon was unable to identify the root canals.

Upon intraoral examination in the area of concern, no sensitivity was observed during horizontal and vertical percussion tests. Probing pocket depth (PPD) was determined to be in the physiologic range. Upon radiographic examination, a small radiolucent area in the furcal region of tooth number 26. Temporary restorative material was removed, revealing a bigger defect area than indicated in the radiographs. Initially, bleeding from the furcal area was controlled with a hemostatic agent-impregnated cotton pack but was not successful and was controlled through extensive irrigation with 2.5% sodium hypochlorite (NaOCl). Working lengths were established using the Root ZX mini Apex locator. The mesial and distal root canals were thoroughly cleaned and shaped with the Woodpecker Gold file system in a crown-down technique, accompanied by irrigation with 3% NaOCl.

Following canal preparation, obturation was done using gutta-percha points and a resin-based sealer (Dia-ProSeal). The perforation site was sealed with a mixture of MTA and sterile saline liquid in a 3:1 ratio. MTA application was facilitated using a curved MTA carrier, and a cotton pellet was subsequently placed in the pulp chamber to create a humid environment conducive to MTA solidification. Finally, the tooth was temporarily filled with Cavit temporary restoration material, ensuring the integrity of the treatment until the next appointment. The patient was recalled to the clinic after three days for reevaluation and reported no symptoms or indications of discomfort. Subsequently, the temporary restorative material along with the damp cotton pellet was carefully removed. An operative explorer was utilized to assess the hardness of the MTA in the treated area.

**Figure 1:**
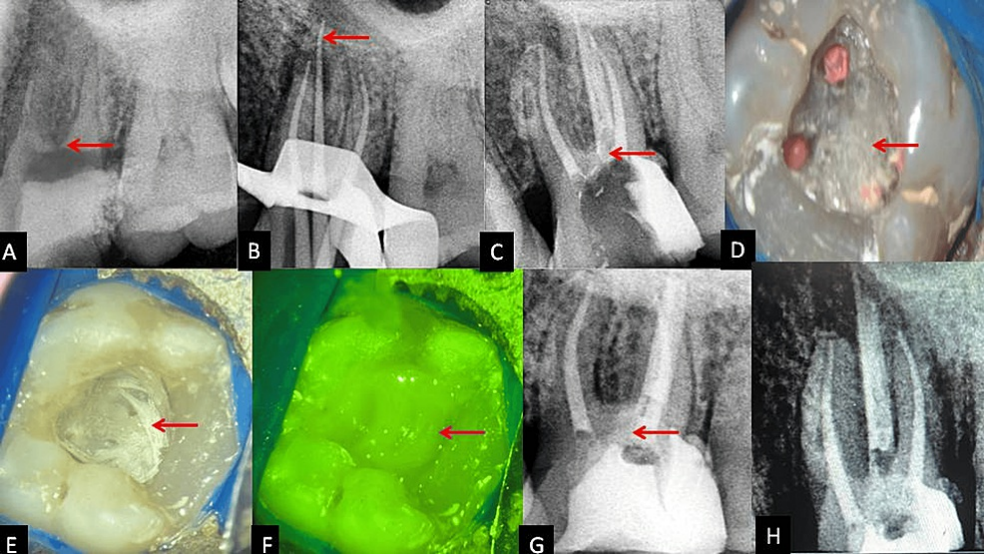
Radiograph images (A) Preoperative radiograph, (B) mastercone radiograph, (C) perforation repair and obturation radiograph, (D) clinical Image after perforation repair, (E) braided fiber placement in the cavity, (F) packable composite placement above glass fibers, (G) immediate postoperative radiograph, (H) six-month follow-up radiograph

On the second visit, the perforation repaired site was covered with type II glass ionomer cement (GIC). Canal orifices were sealed with flowable GIC (SafeEndo ReGlass LC) above etching with 37% phosphoric acid, followed by washing with copious water. The chamber was blotted dry, and a bonding agent was applied after 2% chlorhexidine pretreatment. A 2 mm thickness of flowable composite (3M Espe Filtek Supreme) was applied, and a braided glass fiber impregnated with a light cure composite resin was incorporated into the flowable composite matrix. After light curing this increment with fibers, packable composite (Spectrum, Dentsply) was placed above this increment with tooth number 26. Figure 1 shows the preoperative, intraoperative, postoperative, and six-month follow-up radiographs. 

## Discussion

Endodontic perforations present unique challenges, particularly in the furcal area, where the risk of fracture looms large. While traditional repair materials like MTA have demonstrated efficacy, recent advancements in materials science have introduced fiber-reinforced composites (FRCs) as a promising alternative for reinforcement. FRCs offer a myriad of advantages, including superior mechanical properties, high tensile strength, and modulus of elasticity, rendering them ideal for fortifying weakened tooth structures. Their ability to adapt to irregular cavity shapes and form strong bonds with dentin ensures long-term stability and optimal sealing of perforations, thereby enhancing treatment outcomes.

Glass fiber-reinforced composite (GFRC) stands out as a commonly used reinforcement in dental composites, owing to its exceptional mechanical properties and aesthetic appeal. GFRCs find widespread applications in endodontic therapy, ranging from fabricating posts and cores for endodontically treated teeth to splinting mobile teeth in periodontal therapy. The incorporation of fibers within FRCs plays a pivotal role in modulating stress distribution by forming a monoblock effect, thereby reducing the risk of crack formation and enhancing the overall structural integrity of dental restorations [8,9].

Core materials employed in FRCs, such as polyethylene fibers, glass fibers, and short FRCs, offer unique advantages tailored to specific clinical scenarios. Composites reinforced with polyethylene fibers exhibit excellent stress-modifying properties, dispersing and transferring stresses effectively to mitigate the risk of structural failures [9]. On the other hand, glass fibers provide both reinforcement and aesthetic benefits, enhancing the visual appeal of dental restorations while bolstering their mechanical strength. Furthermore, the choice of restorative material following endodontic treatment plays a pivotal role in preventing tooth fractures and ensuring long-term durability [10].

Recent studies, including research conducted by Luthria et al., have provided valuable insights into the fracture resistance of endodontically treated teeth (ETT) restored with FRCs [11]. Their findings underscored the superiority of glass FRCs over polyethylene FRCs in enhancing fracture resistance, attributing to better wetting and chemical bonding between the fibers and veneering material. The pre-impregnation of glass FRCs further accentuated their reinforcing effect, highlighting the importance of optimizing FRC formulations to maximize clinical outcomes [12].

FRCs represent a significant advancement in reinforcing endodontic perforation repair, offering clinicians a reliable and durable solution for challenging cases. MTA, while established as the gold standard material for perforation repair, can be complemented by FRCs to enhance treatment outcomes and ensure long-term success. However, further research is warranted to refine FRC formulations, standardize clinical protocols, and optimize their performance in perforation repair sites. By harnessing the synergistic properties of MTA and FRCs, clinicians can elevate the standard of care in endodontic therapy and deliver superior outcomes for their patients.

## Conclusions

In the quest of treating teeth with endodontic mishaps, MTA emerges as the unsung hero; MTA seals the breach with bioactive properties. Furthermore, the incorporation of FRCs to MTA fortifies the success of treatment, offering reinforcement to endodontic repair sites and enhancing long-term stability. This synergy between MTA and FRCs ensures a brighter and healthier future in endodontic therapy.
